# Distinct plasma proteome signature at 3 months post-COVID-19 infection irrespective of post-COVID condition

**DOI:** 10.1038/s41598-026-46180-y

**Published:** 2026-04-17

**Authors:** Mohammad Mobarak H. Chowdhury, Akouavi Julite Irmine Quenum, Christine Rioux-Perreault, Jean-François Lucier, Subburaj Ilangumaran, Alain Piché, Hugues Allard-Chamard, Sheela Ramanathan

**Affiliations:** 1 Departments of Microbiology and Infectious Diseases, Faculty of Medicine and Health Sciences, Université de Sherbrooke, Sherbrooke, QC J1H 5N4 Canada; 2 Centre de Recherche du Centre Hospitalier Universitaire de Sherbrooke (CRCHUS), Sherbrooke, QC Canada; 3 Department of Biology, Faculty of Science, Université de Sherbrooke, Sherbrooke, QC J1K 2R1 Canada; 4 Departments of Immunology and Cell Biology, Faculty of Medicine and Health Sciences, Université de Sherbrooke, Sherbrooke, QC J1H 5N4 Canada; 5 Departments of Medicine, Faculty of Medicine and Health Sciences, Université de Sherbrooke, Sherbrooke, QC J1H 5N4 Canada

**Keywords:** Long COVID, Post COVID-19 condition, Oxidative stress, Glutathione, Plasma proteomics, Biochemistry, Biomarkers, Diseases, Medical research

## Abstract

Persistent symptoms following SARS-CoV-2 infection are the hallmark of post-COVID condition (PCC), also referred to as long COVID. However, the underlying molecular mechanisms remain poorly understood. In this study, we employed data-independent acquisition mass spectrometry (DIA-MS)-based plasma proteomics to identify molecular alterations associated with PCC. DIA-MS proteomic analysis revealed a clear distinction between the plasma proteome of uninfected individuals and those previously infected with SARS-CoV-2, irrespective of PCC status. PCC samples demonstrated downregulation of the antioxidant protein peroxiredoxin 6 (PRDX6) and upregulation of oxidative stress–associated proteins, particularly vanin-1 (VNN1) and paraoxonase-3 (PON3). Additionally, individuals with PCC exhibited significantly elevated levels of six proteins—PCSK9, CST3, C1Q, CPB2, KNG1, and GAPDH—associated with glycolysis, complement and coagulation cascades, and inflammatory pathways. Validation by ELISA does not necessarily reflect the proteomics data suggesting the requirement for alternate methods of validation. Nonetheless, oxidative stress, as measured by 8-hydroxy-2′-deoxyguanosine (8-OHdG), further showed that PCC samples had significantly higher levels of DNA damage, compared with convalescent individuals. Antioxidant markers, including reduced and oxidized glutathione (GSH and GSSG), were significantly lower in PCC samples than in uninfected controls. Collectively, these findings indicate that plasma proteomic alterations persist for at least 3 months following SARS-CoV-2 infection, with additional disruptions in oxidative stress and inflammatory pathways characterizing individuals with PCC.

**Supplementary Information:**

The online version contains supplementary material available at 10.1038/s41598-026-46180-y.

## Introduction

Although most COVID-19 infections are relatively mild and resolve within 2–3 weeks^1,2^, a substantial proportion of patients experience a wide range of persistent symptoms following the acute SARS-CoV-2 infection. These symptoms—collectively referred to as *post-COVID-19 condition* (PCC), or “long COVID”—significantly compromise health-related quality of life and impair daily functioning^3–6^. PCC is estimated to affect approximately 65 million individuals worldwide^7,8^, including children^9–12^, and can last for months or even years^13^. PCC comprises a heterogeneous spectrum of clinical manifestations involving multiple organ systems and persisting for more than 2 months after the initial infection has resolved^9,14–20^. Symptoms include, but are not limited to, dysautonomia presenting with cardiac (e.g., palpitations, syncope, dysrhythmias, and orthostatic intolerance), neuropsychiatric (e.g., insomnia, chronic headache, cognitive impairment or “brain fog,” memory deficits, mood disturbances, and pain syndromes), and respiratory (e.g., dyspnea and cough) features)^7,14,21^, as well as generalized asthenia.

PCC has notable impacts on public health, while the understanding of the exact molecular pathways behind its progression are yet poorly characterized. Nonetheless, oxidative stress and abnormal immune response are now recognized as important variables among several possible pathogenic components that may be crucial in controlling the chronic and persistent challenges observed in PCC individuals^22,23^. Reactive oxygen species (ROS) induce lipid peroxidation, protein degradation, and DNA strand damage, all of which can severely disrupt cellular metabolism. Reports indicate that COVID-19 non-survivors exhibit reduced levels of major antioxidants, including vitamin E and glutathione (GSH), whereas survivors show unexpectedly lower levels of superoxide dismutase (SOD) alongside elevated advanced oxidation protein products (AOPP), despite no significant changes in 8-OHdG or malondialdehyde (MDA) levels^24,25^. Moreover, several studies have documented heightened markers of oxidative stress during acute COVID-19, as well as increased concentrations of MDA—a key indicator of lipid peroxidation—in individuals with post-COVID-19 condition^22,23^. Elevated redox stress has been closely associated with a variety of clinical complications, including metabolic syndrome, pulmonary dysfunction, cardiovascular disease, and neurological disorders, all of which are commonly reported in both severe COVID-19 and PCC^26^. However, it remains unclear whether increased ROS levels are direct drivers of PCC symptoms or whether the infection itself, or underlying sub-clinical conditions are responsible for elevating oxidative stress.

In this study, we analyzed the plasma proteome of convalescent, PCC, and uninfected individuals 3 months after PCR-confirmed mild SARS-CoV-2 infection that did not require hospitalization. Our findings indicate that alterations in the plasma proteome persist for at least 3 months following mild infection, and suggest that oxidative stress remains elevated even in individuals who have clinically recovered from SARS-CoV-2 infection.

## Materials and methods

### Participants recruitment

This study used data and plasma samples from the Biobanque Québécoise de la COVID-19 (BQC19), an ongoing provincial healthcare initiative in Quebec, Canada. BQC19 is designed to collect biological specimens—including blood cells and plasma—along with detailed anthropometric and clinical information from individuals diagnosed with SARS-CoV-2 infection confirmed by PCR testing. Since March 26, 2020, the initiative has recruited adult participants (18 years and older) with varying degrees of disease severity from multiple healthcare centers across Quebec^27^. For the present study, samples were obtained from the Université de Sherbrooke Hospital Research Center between March 2020 and October 2021, and all infections were confirmed before October 2021. The study design, data collection methods, and procedures have been described in detail elsewhere^28^. SARS-CoV-2 infection severity was classified according to the World Health Organization (WHO) criteria as asymptomatic, mild, moderate, or severe^29^. The study received approval from the ethics review board of the Centre de Recherche du Centre Hospitalier Universitaire de Sherbrooke (protocol #2022-4415).

### Application of mass spectrometry for protein analysis

#### Methods for protein preparation and protease digestion prior to mass spectrometry

Plasma samples were prepared for proteomic analysis by the proteomics platform at Université de Sherbrooke following established protocols as described in Chowdhury et al.^30^. All experimental methods were performed in accordance with the relevant guidelines and regulations. In brief, after determining the protein concentration by Lowry protein Assay in the plasma. 75 ug of proteins were reduced in 50 μL of 10 mM HEPES–KOH pH 7.5, 8 M urea containing Dithiothreitol (DTT) to a final concentration of 5 mM followed by heating at 95 °C for 2 min, and a 30-min incubation at room temperature. Chloroacetamide (ClAA) (Sigma-Aldrich, Saint-Louis) was added to a final concentration of 7.5 mM to alkylate the proteins followed by a 20-min incubation at room temperature in the dark. The urea was diluted to a final concentration of 2 M by adding 50 mM ammonium bicarbonate (NH_4_HCO_3_) (Sigma-Aldrich, Saint-Louis). The proteins were digested by adding 1 μg of Pierce MS-grade trypsin (Thermo Fisher Scientific, Waltham) and incubated overnight at 30 °C with shaking. Peptides were purified with micropipette tips containing a C18 column (EMD Millipore, Burlington, VT), concentrated by centrifugal evaporator and then resuspended in formic acid buffer. Peptides were assessed using a NanoDrop spectrophotometer (Thermo Fisher Scientific, Waltham, MA).

For DIA LC–MS analysis, 250 ng of peptides from each sample were injected into an HPLC (nanoElute, Bruker Daltonics) and loaded onto a trap column with a constant flow of 4 µL/min (Acclaim PepMap100 C18 column, 0.3 mm id × 5 mm, Dionex Corporation) then eluted onto an analytical C18 Column (1.9 µm beads size, 75 µm × 25 cm, PepSep) heated at 50 °C. Peptides were eluted over a 2-h gradient of ACN (5–37%) in 0.1% FA at 400 nL/min while being injected into a TimsTOF Pro ion mobility mass spectrometer equipped with a Captive Spray nano electrospray source (Bruker Daltonics). Data was acquired using diaPASEF mode. Briefly, for each single TIMS (100 ms) in diaPASEF mode, we used 1 mobility window consisting of 27 mass steps (m/z between 114 and 1414 with a mass width of 50 Da) per cycle (1.27 s duty cycle). These steps cover the diagonal scan line for + 2 and + 3 charged peptides in the m/z-ion mobility plane.

#### Analysis and visualization of PCA and differentially expressed proteomic data

Raw data files were processed using the DIA-NN software v.1.8.1 (DIA-NN: neural networks and interference correction enable deep proteome coverage in high throughput)^31^ available on GitHub (https://github.com/vdemichev/DiaNN). DIA-NN was installed in an Apptainer container using the Docker image from Docker Hub (https://hub.docker.com/layers/biocontainers/diann/v1.8.1_cv1/images). The analysis was conducted with default parameters. The human proteome (FASTA file UP000005640) was obtained from the Uniprot database https://ftp.uniprot.org/pub/databases /uniprot/current release/knowledgebase/referenceproteomes/Eukaryota/UP000005640/), comprising 96,418 protein entries. For the FASTA-based searches, DIA-NN was configured to conduct in-silico digestion of the protein sequences. A mass tolerance of 20 ppm was applied for both MS1 precursor ions and MS2 fragment ions. The peptide length was limited to 7–30 amino acids, and precursor charge states were set between 1 and 5. The precursor m/z range was configured from 100 to 1700, and fragment m/z from 100 to 1500. These parameters were employed for both library generation through in-silico approaches and library-free searches. Match Between Runs (MBR) and smart profiling functionalities were activated to construct a spectral library directly from the DIA data. We used –unimod4 (Carboxyamidomethylation)–unimod35 (Oxidation or Hydroxylation) as fixed modifications, while N-terminal protein acetylation was treated as a variable modification. Post-analysis was conducted using R (version 4.3.2; www.R-project.org), resulting in the final output file titled “unique_genes_matrix.tsv”. This matrix contained quantified gene-specific peptides filtered using a global false discovery rate (FDR) of 1%, applying both global q-values for protein groups and global as well as run-specific q-values for precursors. Twenty repeated samples were utilized solely for validation purposes and were excluded from statistical analyses; consequently, data from 150 unique samples were included in the final analysis. The dataset can be accessed at PRIDE-pxd066724.

#### Protein validation by ELISA

Protein levels for GAPDH (Catalog # EH207RB), Proprotein Convertase 9/PCSK9 (Catalog # EH384RB), sCD26 (Catalog # BMS235), Carboxypeptidase B2/CPB2 (Catalog # EH68RB), and C1q (Catalog # BMS2099 or BMS2099TEN) in plasma were quantified according to the specific protocols provided by the manufacturers (ThermoFisher, USA). CST3 (Cystatin C) ELISA kit was obtained from Sinobiologicals (# sek10439).

#### Gene ontology and pathway enrichment analyses

To explore the functional characteristics of common proteins between PCC, convalescent and uninfected groups, we performed the comprehensive enrichment analyses employing the SRplot web server, a robust web-based gene set enrichment tool (http://www.bioinformatics.com.cn/srplot). Visualization and further analysis of the resulting protein–protein interaction (PPI) networks were performed using Cytoscape software (version 3.8.2). Furthermore, we employed the cytoHubba plugin within Cytoscape for applying the degree of topological algorithm. This approach allowed us to select the most critical hub proteins with the highest degree of connectivity which are potentially significant in the underlying biological processes.

### Antioxidant glutathione (GSH + GSSG) measurement in plasma by colorimetric assay

Plasma samples isolated from fresh blood through centrifugation and stored at − 80. The total levels of glutathione (GSH + GSSG) in plasma were quantified using the Glutathione Colorimetric Detection Kit (Invitrogen, Catalog Number: EIAGSHC). Samples were initially diluted with the Assay Buffer provided in the kit to ensure their concentrations were within the detectable range. To specifically measure GSSG levels, a subset of samples was treated with 2-vinylpyridine (2VP) in separate tubes to block free GSH. For the assay, 50 µL of each diluted sample and standard was dispensed into a 96-well microplate. Subsequently, 25 µL of the colorimetric detection reagent and 25 µL of a reaction mixture containing glutathione reductase solution were added to each well. The plate was then incubated at room temperature for 20 min to allow the reaction to occur. Absorbance was measured at 405 nm using a microplate reader. Separate standard curves were constructed for GSH and for 2VP-treated GSSG using serial dilutions of the Glutathione Standard, which enabled the calculation of GSH and GSSG concentrations in the samples. All assays were conducted in duplicate to ensure accuracy, and the results were analyzed to evaluate oxidative stress levels.

### Oxidative DNA damage and antioxidant protein measurement in plasma by ELISA

The concentration of free 8-hydroxy-2′-deoxyguanosine (8-OHdG) in plasma samples was determined using a competitive ELISA kit provided by Stressmarq Biosciences, Canada (Catalog Number: SKC-120A), following the manufacturer’s protocol as previously described by us^30^. Additionally, the concentrations of Human Cu/ZnSOD in plasma were quantified according to the specific protocols provided by the manufacturers (Invitrogen, USA).

### Statistical analyses

Statistical analyses of oxidative stress and plasma proteomic profiles were conducted using GraphPad Prism version 10.0.3 (San Diego, CA, USA). To ensure baseline similarities and make comparisons between groups, an independent t-test was employed. P-values were determined using one-way ANOVA followed by Tukey’s multiple comparison test while keeping the threshold for statistical significance at *p* ≤ 0.05. The results are expressed as the mean ± standard deviation (SD). GraphPad Prism was employed to create volcano plots and quantify protein abundance between the study groups. Venn diagrams were generated using the jvenn online tool (https://jvenn.toulouse.inrae.fr/app/index.html) and DIA-NN software. Furthermore, PCA was conducted to cluster peptide-based intensity and intact protein detection across three groups using ELISA. The analysis was performed with the SRplot web server (https://www.bioinformatics.com.cn/en).

## Results

### Baseline characteristics of the cohort studied

Samples collected during the initial phase of the pandemic (2020–2021) were included in this study as well as in the previous study^28^ (Table 1). Plasma samples were obtained from individuals attending the COVID-19 clinic, where SARS-CoV-2 infection was confirmed by RT-PCR as previously described^27^. Uninfected controls (Un-inf) were SARS-CoV-2 RT-PCR–negative at the time of sample collection. Participants in the convalescent (Conv) and post-COVID condition (PCC) groups were PCR-negative 1 month after infection. Individuals in the Conv group were free of infection-related symptoms at 3 months post-infection, whereas those in the PCC group reported persistent symptoms consistent with WHO criteria in effect during the sample collection period^29^.

**Table 1:** Baseline clinical characteristics of study group.

Variables	No infection n = 35	Convalescent n = 62	PCC n = 53
Female SEX			
n (%)	21 (60.0)	26 (41.9)	34 (64.1)
Age range	18–73	18–81	18–88
Comorbidities (n)			
Obesity		9 (15.6)	9 (15.6)
Hypertension	0	1	5
Diabetes	1	4	7
Asthma	1	2	6
Number of PCC symptoms at 3 months post-infection range (mean)	0	0	1–16 (2.9 ± 2.8)

PCC—convalescent individuals with PCC; *PCC* post-COVID condition.

### Proteomics analysis

#### DIA-MS-based differentially expressed proteins and PCA analysis

**Fig. 1:**
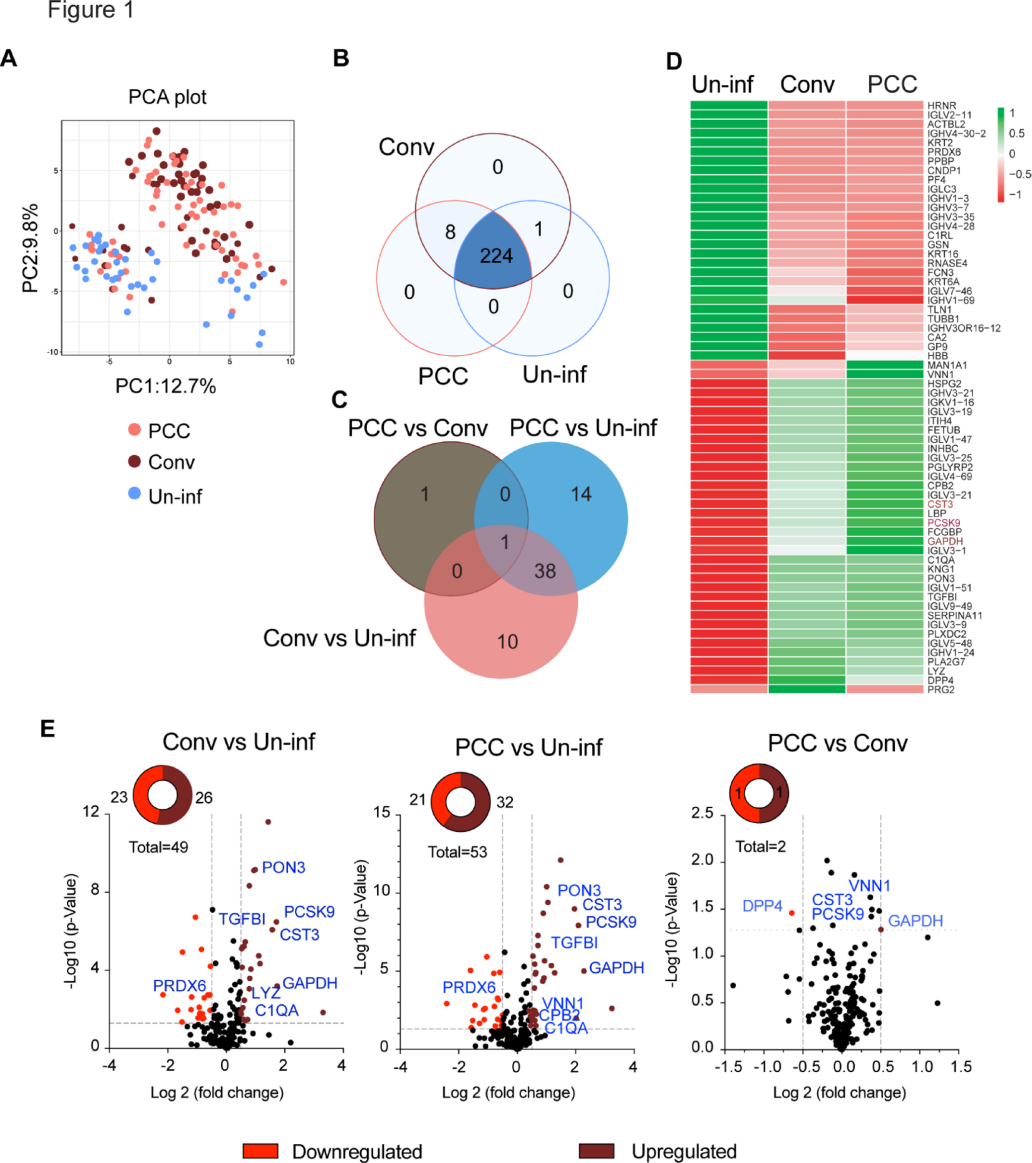
Proteomic analyses. (**A**) PCA plot analysis. The PCA plot separated the groups into two principal components, PC1 and PC2. Each dot represents an individual sample in the PCA plot. (**B**) Venn diagram represents each group’s shared common protein and unique proteins, with the overlapping region denoting common proteins across all three conditions. The DIA-analyst application platform (Analyst Suites) was used for PCA and Venn diagram analyses. (**C**) The Venn diagram shows unique and common significant differential proteins in PCC, convalescent and un-infected individuals, with the central overlap representing the proteins shared among the groups. (**D**) Comprehensive Heatmap analysis for differentially expressed peptide abundance profiles. (**E**) Comparative Volcano plot analysis of plasma protein expression among three groups. Proteins that are significantly modulated (fold change of ± 0.5 and *p* value of 0.05, indicated by dotted line) are labeled and shown in red and green color. The x-axis represents the log2 fold change, and the y-axis displays the − log10 *p* value.

We used a data-independent acquisition (DIA) proteomics approach to analyze plasma collected at 3 months post-PCR-confirmed SARS-CoV-2 infection from 53 individuals with PCC, 62 recovered individuals without PCC, and 35 healthy controls (SARS-CoV-2 PCR-negative). A total of 235 unique proteins were identified across the three groups and quantified using DIA-NN, focusing on prototypic peptides uniquely mapped to individual proteins (Supplementary Table 1). Principal component analysis (PCA) (Fig. 1A) showed that both convalescent and PCC groups were clearly separated from uninfected controls; however, the convalescent and PCC groups displayed minimal separation, indicating limited differences in their protein expression profiles. This trend was consistent with the Venn diagram (Fig. 1B), which showed that 224 proteins were shared among all groups, with only eight proteins shared exclusively between the convalescent and PCC groups and one shared between the PCC and uninfected groups. Overall, there was broad overlap in differentially abundant proteins (DEPs) between the PCC and convalescent groups when each was compared with uninfected individuals, suggesting that proteomic changes induced by mild COVID-19 persist for at least 3 months but do not distinguish convalescent individuals from those with PCC (Fig. 1C). Heatmap analysis further differentiated uninfected individuals based on their unique peptide expression patterns, yet it did not separate PCC from convalescent individuals (Fig. 1D, Supplementary Fig. 1). Differences observed in Ig-H, Ig-λ, and Ig-κ peptides may reflect variations in immunoglobulin usage associated with the antibody response to SARS-CoV-2. DEP analysis identified 49 proteins that were differentially abundant in the convalescent group relative to uninfected controls, including 26 upregulated and 23 downregulated proteins (Fig. 1E, left panel). Similarly, 53 proteins were differentially expressed between PCC and uninfected individuals, while only 2 proteins differed between PCC and convalescent individuals (Fig. 1E, middle and right panels).

**Fig. 2:**
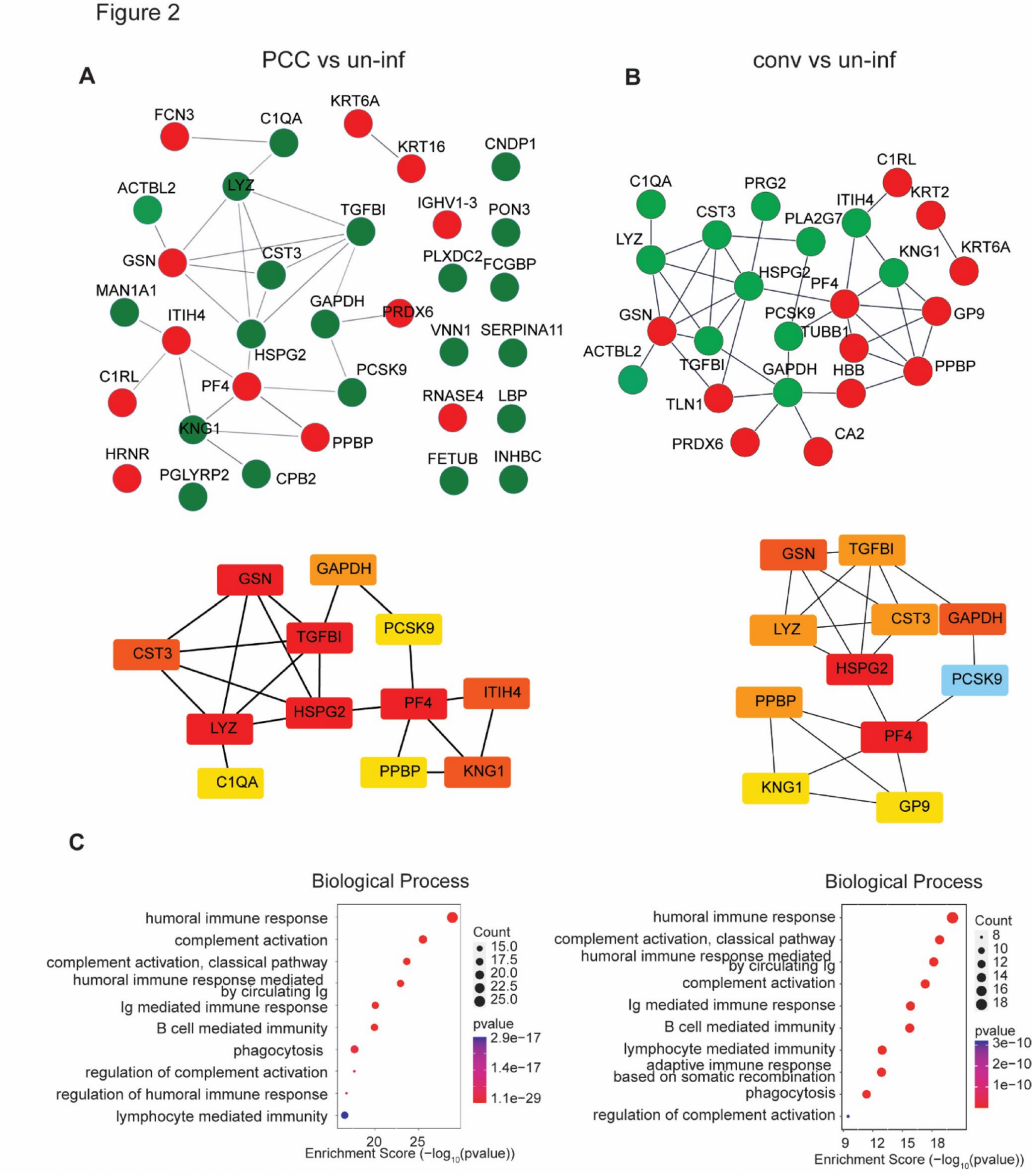
Protein–protein interaction network (PPI) analysis for the differential expressed protein. (**A**,**B**) PPI network illustrates significant differentially expressed proteins (24) between PCC (**A**), convalescent (**B**) and uninfected individuals. Lower panel highlights the top ten hub proteins identified by CytoHubba in Cytoscape based on the degree of connectivity of each protein node. In this network, nodes represent individual proteins, whereas edges indicate their interactions. The green and red nodes indicate the upregulated and downregulated proteins, respectively. Red, orange, yellow and blue represent the degree of connectivity from high to low. The PPI network was constructed using the STRING database version 12.2 and visualized with Cytoscape 3.10.2. (**C**) Gene ontology analysis for the biological process between convalescent group vs un-infected group (left panel), and PCC group vs un-infected group (Right panel). Dot plots illustrate the number of proteins associated with each GO biological term and their corresponding significance (*p* value). The analysis was conducted using the SR plot web server, with GO terms considered significant at a *p* value < 0.05.

Protein–protein interaction (PPI) analysis using the STRING database (version 12.2) revealed that eight differentially expressed proteins between PCC and uninfected individuals formed a single interaction network, while thirteen proteins showed no detectable interactions (Fig. 2A). Nineteen (19) differentially expressed proteins showed at least one interaction in the STRING analysis, while the remaining thirteen differentially expressed proteins showed no direct interactions. The resulting network was further analyzed in Cytoscape using the CytoHubba plugin to identify the most influential proteins based on degree centrality. The top 12 hub proteins encompassed diverse functional categories, including oxidative stress and metabolic activity (GAPDH), inflammation (CST3, TGFB1), and complement and coagulation pathways (C1QA, KNG1) (Fig. 2B).

Similarly, analysis of differentially expressed proteins between convalescent and uninfected individuals identified a single interaction cluster comprising 24 proteins (Fig. 2B). CytoHubba identified eleven top hub proteins in this comparison (Fig. 2A), most of which overlapped with those identified in the PCC network. Collectively, these findings indicate that at 3 months post-infection, individuals with prior SARS-CoV-2 infection exhibit broadly similar proteomic signatures, regardless of PCC status.

GO enrichment analysis of the differentially expressed proteins among the PCC, convalescent, and uninfected groups revealed enrichment of terms associated with immune responses (Fig. 2C). Enrichment of “humoral immune response” (GO:0006959) and “immunoglobulin-mediated immune response” (GO:0016064) likely reflects differences in antigen receptor profiles arising from immune responses generated against SARS-CoV-2 infection. Although the persistence of antibody-related differences at 3 months post-infection is consistent with the long lifespan of circulating antibodies, it was notable that alterations in complement pathways (GO:0006956, GO:0006958) and phagocytosis (GO:0006909) also persisted in both the PCC and convalescent groups. Together, these observations indicate sustained perturbations in immune homeostasis well after viral clearance.

#### Validation of differentially expressed proteins or biomarkers by peptide intensity and ELISA assay quantification

**Fig. 3:**
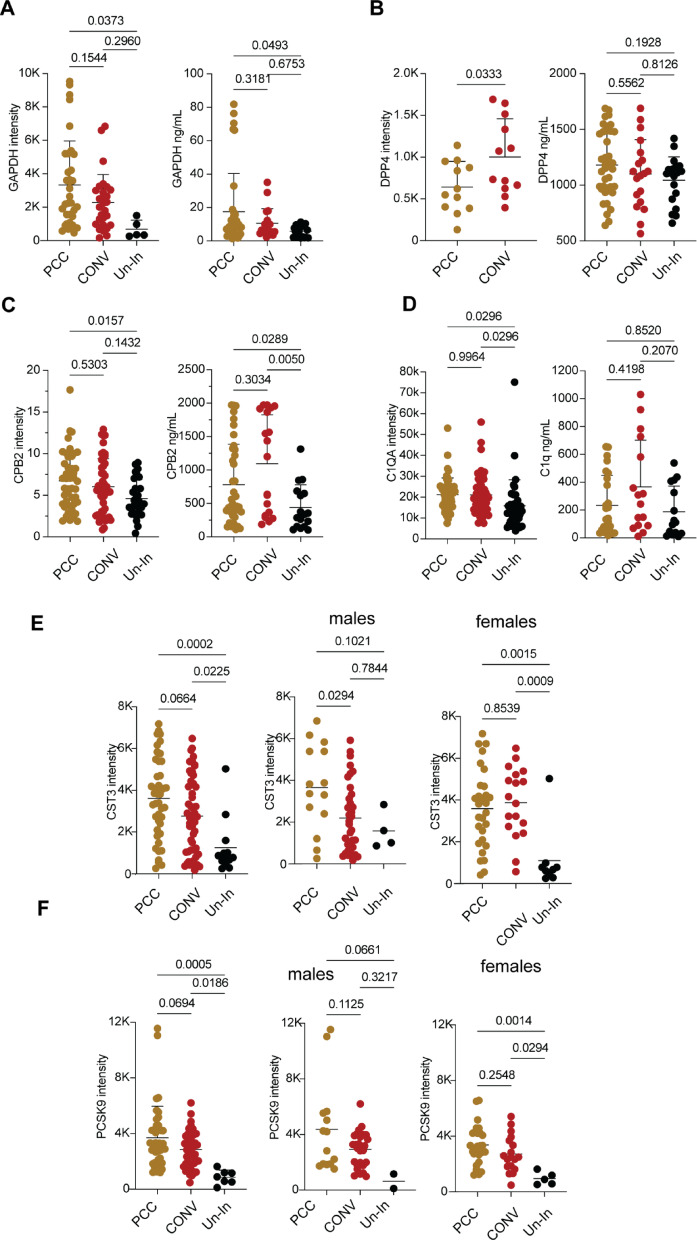
Comparison between proteomics and ELISA measurements for a restricted set of proteins. Peptide abundance (DIA-MS) and protein levels (ELISA) are shown for select proteins across three groups. Statistical analysis was conducted using one-way/two-way ANOVA followed by Tukey’s multiple comparisons tests, with significance set at a *p* value ≤ 0.05. Data are presented as mean with standard deviation.

To validate the differentially expressed proteins identified in plasma samples across the three groups, we selected CST3, GAPDH, PCSK9, C1QA, CPB2, and PRDX6 based on significant fold changes and pathway enrichment scores. Because DIA-NN quantifies peptide intensities in an unbiased manner at the gene-group level, we sought to confirm the presence of epitopes corresponding to these peptides using ELISA. For this validation, we randomly selected 40 PCC, 20 convalescent, and 20 uninfected individuals and used commercially available ELISA kits. GAPDH peptide intensity was significantly elevated in PCC individuals but not in convalescent individuals. This trend was reproduced in ELISA-based detection of GAPDH epitopes (Fig. 3A). DPP4 was not detected in uninfected samples by proteomics; however, a subset of convalescent and PCC samples showed detectable levels, with significant differences between the two infected groups (Fig. 3B). By contrast, ELISA detected DPP4 in all three groups with comparable levels. As a circulating serine protease are involved in inflammation and immune regulation, DPP4 detection across groups is expected.

Three proteins associated with complement and coagulation pathways—C1QA, CPB2, and KNG1—showed significant differences in PCC individuals by proteomics. CPB2, a regulator of fibrinolysis, was significantly elevated in PCC compared with uninfected individuals by both proteomics and ELISA (Fig. 3C). While CPB2 peptide abundance mirrored the GAPDH pattern, ELISA showed that both PCC and convalescent groups were significantly higher than uninfected controls. Similarly, peptide abundance for C1QA was significantly increased in the PCC and convalescent groups, although ELISA measurements were comparable across groups (Fig. 3D). Stratification of data by biological sex suggested potential sex-based differences; however, the limited sample size prevents definitive conclusions, and these observations should be interpreted cautiously (Supplementary Fig. 2).

Cystatin C (CST3) and proprotein convertase subtilisin/kexin type 9 (PCSK9), both previously associated with COVID-19 pathology^32–34^, also showed divergent trends between proteomics and ELISA. CST3 was strongly upregulated in both PCC and convalescent groups by proteomics (Fig. 3E), whereas ELISA revealed reduced CST3 levels in these groups compared with uninfected controls (Supplementary Fig. 3A). Conversely, PCSK9 peptide abundance was significantly elevated in PCC and convalescent groups, but ELISA measurements did not reflect these differences (Fig. 3F; Supplementary Fig. 3B). The discrepancies between peptide abundance and ELISA measurements for CST3 and PCSK9 may reflect post-translational modifications, proteolytic processing, or conformational changes affecting epitope accessibility for antibody-based detection, despite increased peptide-level representation detected by mass spectrometry.

**Fig. 4:**
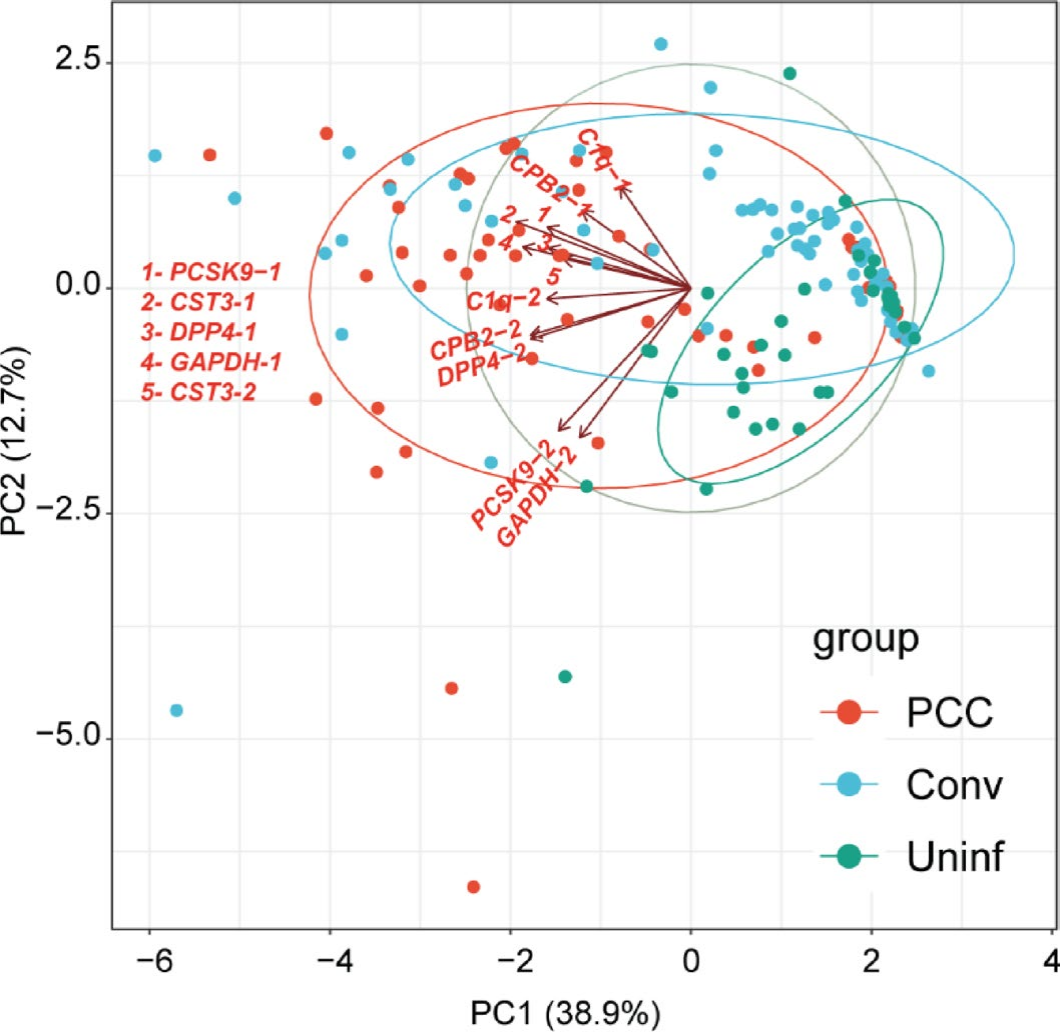
Validation of differential expressed protein by PCA. The PCA plot shows the separation of three groups. The ellipses provide a visual sense of how much variability exists within each group and how much overlap (similarity) or separation (difference) there is between the groups. Less overlap suggests a more distinct separation between the groups, while more overlap suggests similarity. Arrows indicate the weightage of peptides relevant to proteins such as PCSK9, GAPDH, DPP4, CPB2, and CST3 contributing to the separation of the groups along the principal components (PC1 & PC2). In the PCA plot, 1 = Peptide intensity by DIA-MS; and 2 = intact proteins by ELISA assay. The gray ellipse indicates the 95% confidence region of the data distribution in PC1–PC2 space.

We integrated the proteomics and ELISA datasets to assess whether the PCC group could be distinguished from the convalescent group using PCA (Fig. 4). The PCA plot combines peptide intensities and ELISA-based protein measurements into a two-dimensional representation, enabling visualization of similarities and differences across the three groups. Six proteins measured by ELISA (GAPDH, PCSK9, DPP4, CPB2, C1QA, and CST3) were included in this analysis. PCC, convalescent, and uninfected groups showed partially overlapping but distinguishable proteomic profiles. The uninfected controls formed a tight, well-defined cluster in the right quadrant, indicating low intra-group variability and clear separation from the infected groups. The convalescent group overlapped partially with both PCC and uninfected individuals, suggesting an intermediate molecular profile. In contrast, the PCC group formed a distinct cluster toward the left side of the plot, indicating a proteomic signature different from both the convalescent and uninfected groups. Arrows represent the direction and magnitude of each protein’s contribution to sample separation. Longer arrows indicate stronger influence on group clustering, while direction suggests association with specific groups. GAPDH, PCSK9, CST3, CPB2, and C1QA all point toward the PCC cluster—particularly their ELISA-derived features (indicated by the “2” suffix)—suggesting higher expression levels or greater discriminatory power in PCC individuals. Notably, ELISA-based and MS-based detections generally aligned in directionality, indicating good concordance between the two platforms.

### Analysis of antioxidant activity and DNA-damaged oxidative stress

**Fig. 5:**
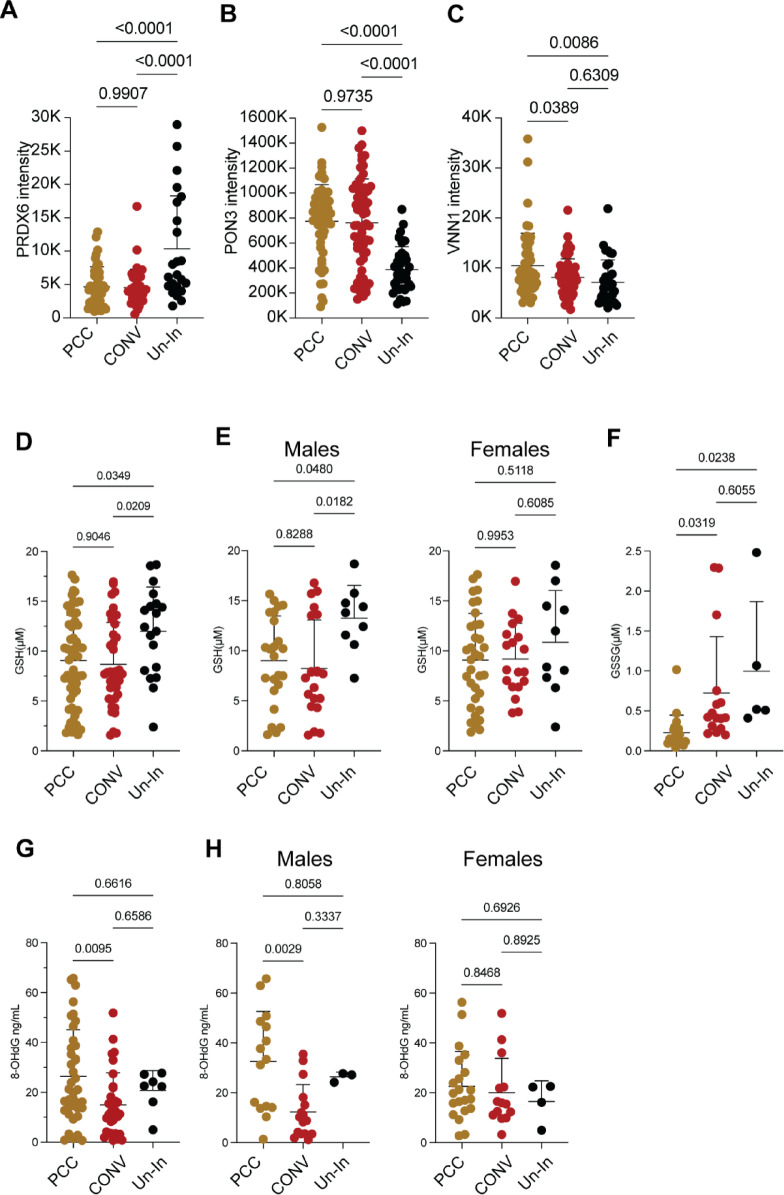
Expression levels of peptides and markers of oxidative stress in plasma. (**A**–**C**) Peptide abundance (DIA-MS) for PRDX6, PON3 and VNN; (**D**,**E**) GSH, (**F**) GSSG and (**G**,**H**) 8-OHdG DNA concentrations in the three groups. Statistical comparisons were carried out using one-way ANOVA followed by Tukey’s multiple comparisons test with significance determined at a *p* value ≤ 0.05. The data are presented as mean + standard deviation (SD), and different colors represent this group. *GSH* glutathione, *GSSG* Glutathione disulfide, *8-OHdG* 8-hydroxy-2′-deoxyguanosine, *PCC* post COVID condition, *CONV* convalescent, *Un-In* uninfected.

Several studies have reported an association between SARS-CoV-2 infection and a reduced capacity to manage oxidative stress^25,35^. However, the oxidative stress status in individuals with PCC remains poorly defined^26,36,37^. In our proteomic analysis, we identified several proteins implicated in oxidative stress pathways that were differentially expressed across groups (Fig. 5A, Supplementary Fig. 4). Peroxiredoxin 6 (PRDX6), a bifunctional enzyme with antioxidant activity^38^, was significantly downregulated in both the convalescent and PCC groups compared with uninfected controls. In contrast, paraoxonase-3 (PON3) and Vanin-1 (VNN1)—both known to be upregulated under oxidative stress conditions^39,40^—were significantly increased in the PCC group relative to uninfected individuals (Fig. 5B,C). PON3 is known to protect against mitochondrial oxidative stress^41^, while elevated VNN1 levels are associated with enhanced oxidative stress responses^42^. Together, these findings suggest that oxidative stress induced by SARS-CoV-2 infection persists for at least 3 months post-infection, with PCC individuals showing the strongest molecular signatures of ongoing oxidative stress compared to uninfected controls.

To complement our proteomic analyses, we measured plasma GSH levels at 3 months post-infection in PCC and convalescent groups as an indicator of oxidative stress^43,44^. No significant differences in GSH concentrations were observed between PCC and convalescent individuals (Fig. 5D). However, both groups exhibited significantly reduced GSH levels compared with uninfected controls (*p* ≤ 0.05). When stratified by biological sex, males in both the PCC and convalescent groups showed significantly lower GSH levels than uninfected males (Fig. 5E), whereas no significant differences were detected among females. These findings suggest that GSH depletion occurs during and after mild COVID-19 infection and persists for at least 3 months, regardless of PCC status. In contrast, levels of oxidized glutathione (GSSG) were significantly lower in the PCC group compared with the convalescent group (*p* ≥ 0.05) (Fig. 5F). These data indicate that total GSH and oxidized GSH may follow different recovery trajectories after SARS-CoV-2 infection, potentially diverging between PCC and convalescent individuals. Measurement of the antioxidant enzyme Cu/Zn SOD by ELISA revealed comparable levels across all three groups (Supplementary Fig. 5). To further assess oxidative stress, we quantified circulating 8-hydroxy-2′-deoxyguanosine (8-OHdG), a marker of oxidative DNA damage generated when hydroxyl radicals oxidize guanine residues. 8-OHdG concentrations were significantly elevated in the PCC group compared with the convalescent group (Fig. 5G). When stratified by sex, this increase was significant in males but not females with PCC relative to convalescent individuals, mirroring the pattern observed for GSH (Fig. 5H).

## Discussion

Our study demonstrates that the plasma proteome of SARS-CoV-2–naïve individuals is clearly distinct from that of both convalescent and PCC groups. This finding was unexpected, as we initially anticipated that convalescent individuals would more closely resemble uninfected controls. While several proteomic studies have focused on identifying plasma biomarkers in patients with severe COVID-19^45–51^, relatively few have examined individuals with mild infections—the population that represents the majority of long COVID cases^52^. Our data show that even after mild infection, the plasma proteomic signature of uninfected individuals differs substantially from that of both convalescent and PCC groups. Whether this pattern is unique to SARS-CoV-2 or reflects a broader feature of post-viral recovery remains unknown. Notably, at 3 months post-infection, differences between the convalescent and PCC groups are subtle, suggesting that persistent proteomic alterations occur after mild COVID-19 but may not fully explain PCC-specific symptomatology.

In this study, the value of mass spectrometry–based proteomic approaches for biomarker discovery was reinforced through partial validation using ELISA. The six targeted proteins (GAPDH, PCSK9, DPP4, CPB2, C1QA, and CST3) were differentially expressed in PCC and convalescent groups compared with healthy controls, indicating persistent molecular alterations following SARS-CoV-2 infection. CPB2, a key regulator of fibrinolysis, has previously been associated with thrombotic complications and disease severity in COVID-19^53–55^. Consistent with these reports, both peptide abundance and ELISA validation showed elevated CPB2 in PCC, highlighting its potential involvement in sustained coagulation abnormalities and its promise as a therapeutic target.

GAPDH was also consistently upregulated across all PCC comparisons. Beyond its canonical role in glycolysis, GAPDH is known for its redox sensitivity, involvement in mitochondrial dysfunction, and even antiviral activity through interactions with the SARS-CoV-2 spike protein^56–58^. Our DIA-MS and ELISA results confirmed higher GAPDH levels in PCC, suggesting activation of glycolytic and redox-responsive pathways and pointing toward an immune-metabolic imbalance in individuals with PCC.

Overall, our findings support the robustness of the proteomic dataset, as two of the six proteins—GAPDH and CPB2—showed strong concordance between DIA-MS and ELISA. These consistent biomarkers hold promise for clinical applications, including monitoring PCC progression or guiding therapeutic strategies. In contrast, the remaining proteins exhibited substantial variability between assays, indicating that they may be less reliable as standalone biomarkers and warrant further investigation in larger cohorts. Validation of potential biomarkers by different assays will be necessary to determine the robustness of any biomarker, while the pathways identified can provide information on the identification of alternate targets.

The involvement of oxidative stress in PCC has been reported by multiple groups^22,23,26,59–63^. Lower levels of circulating free thiols—which serve as markers of systemic oxidative stress—have been shown to persist in non-hospitalized individuals who later developed long COVID^23^. Consistent with these findings, our proteomic and biochemical analyses demonstrate that, at 3 months post-infection, both convalescent individuals and those with PCC exhibit alterations in oxidative stress markers that differ significantly from uninfected controls. Longitudinal analyses will be essential to determine whether these markers can reliably predict PCC onset or persistence.

Long COVID, or PCC, encompasses a wide range of symptoms of varying intensity, and these symptoms often show limited correlation with the severity of the initial SARS-CoV-2 infection^64^. Five years into the COVID-19 pandemic, PCC continues to impose a substantial burden on affected individuals, with long-term symptoms compromising quality of life. Longitudinal studies indicate that a greater number of symptoms during the acute infection may serve as a predictor of PCC development^65–67^. We and others have previously shown that PCC is associated with uncoordinated or dysregulated immune responses to SARS-CoV-2 antigens^28,68^, a finding that aligns with reports of altered immune profiles in PCC from other research groups^52^.

Inflammation-related biomarkers and autoantibodies have also been found at elevated levels in PCC patients compared with convalescent individuals recovering from severe infection^49,69^. Collectively, diverse methodological approaches—including proteomics, immunophenotyping, and serological profiling—converge on the conclusion that long COVID is distinguishable by specific biological signatures. However, meaningful clinical application will require integrating these biomarkers with the heterogeneous symptom profiles and phenotypic spectrum that characterize PCC as a complex and multifaceted condition.

### Limitations and strengths of the study and future research

A key limitation of this study is the relatively small sample size. Our cohort was restricted to non-hospitalized individuals infected during the early phase of the pandemic (2020–2021), a period when strict public health measures reduced widespread community transmission and consequently limited recruitment opportunities. Although PCC diagnosis was based on the WHO criteria available in 2020^29^, it is now recognized that PCC incidence and predominant symptom profiles can vary depending on the SARS-CoV-2 variant in circulation at the time of infection^70^. This contextual variability may influence the generalizability of our findings. Despite these limitations, our study provides an important insight: oxidative stress can be effectively monitored using minimally invasive analyses of peripheral blood samples. While additional measurements covering a broader array of oxidative stress markers would have offered a more comprehensive understanding, the current results highlight the feasibility and potential clinical utility of tracking oxidative stress–related pathways in PCC and post-infectious recovery.

## Supplementary Information

Below is the link to the electronic supplementary material.

Supplementary Material 1

## Data Availability

The data can be made available after obtaining relevant authorizations. The mass spectrometry proteomics data have been deposited to the ProteomeXchange Consortium via the PRIDE partner repository with the dataset identifier PXD066724 (https://www.ebi.ac.uk/pride/archive?keyword=pxd066724).
