# Supplementary material for: Distinct plasma proteome signature at 3 months post-COVID-19 infection irrespective of post-COVID condition
Source: Sci Rep. 2026 Apr 17;16:18201. doi: 10.1038/s41598-026-46180-y (PMC13260905; doi:10.1038/s41598-026-46180-y)
Supplement: Supplementary file 1 — Supplementary Material 1 [file 41598_2026_46180_MOESM1_ESM.pdf]

Supplementary figures:

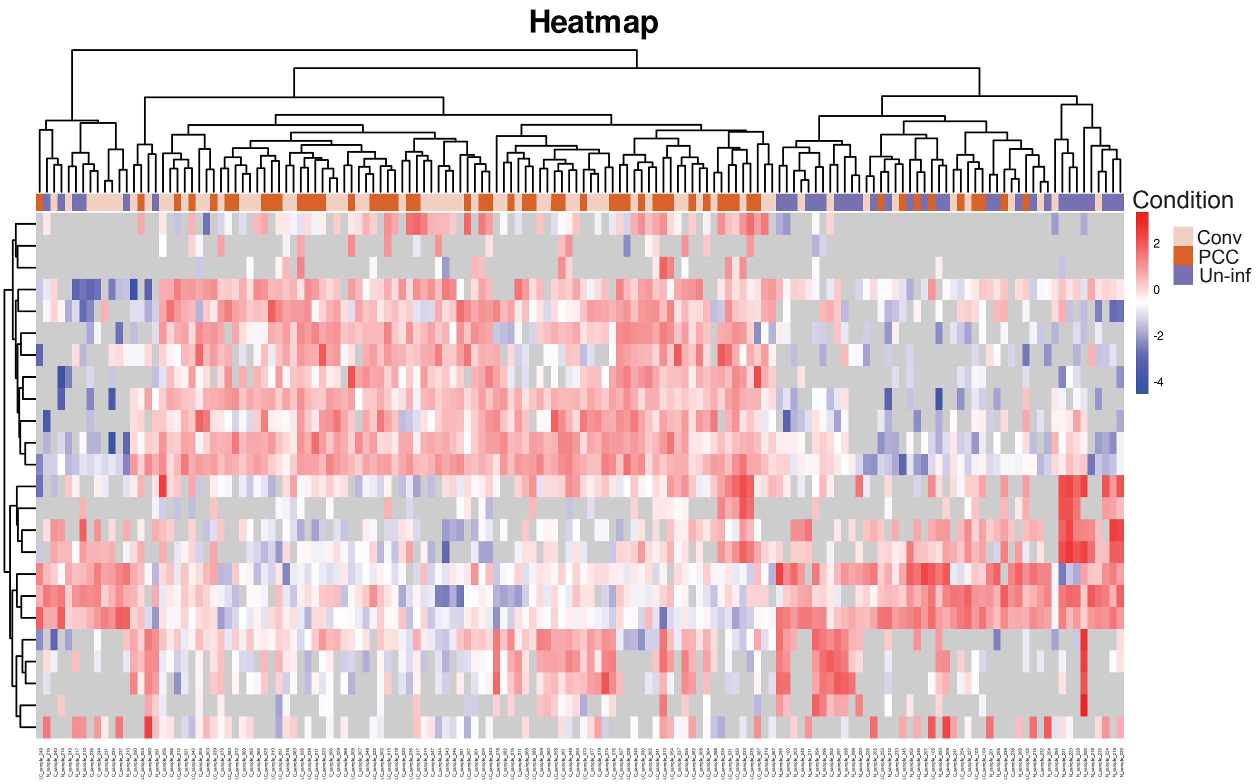

Supplementary Fig. 1 Heatmap of differentially expressed proteins in all the samples.

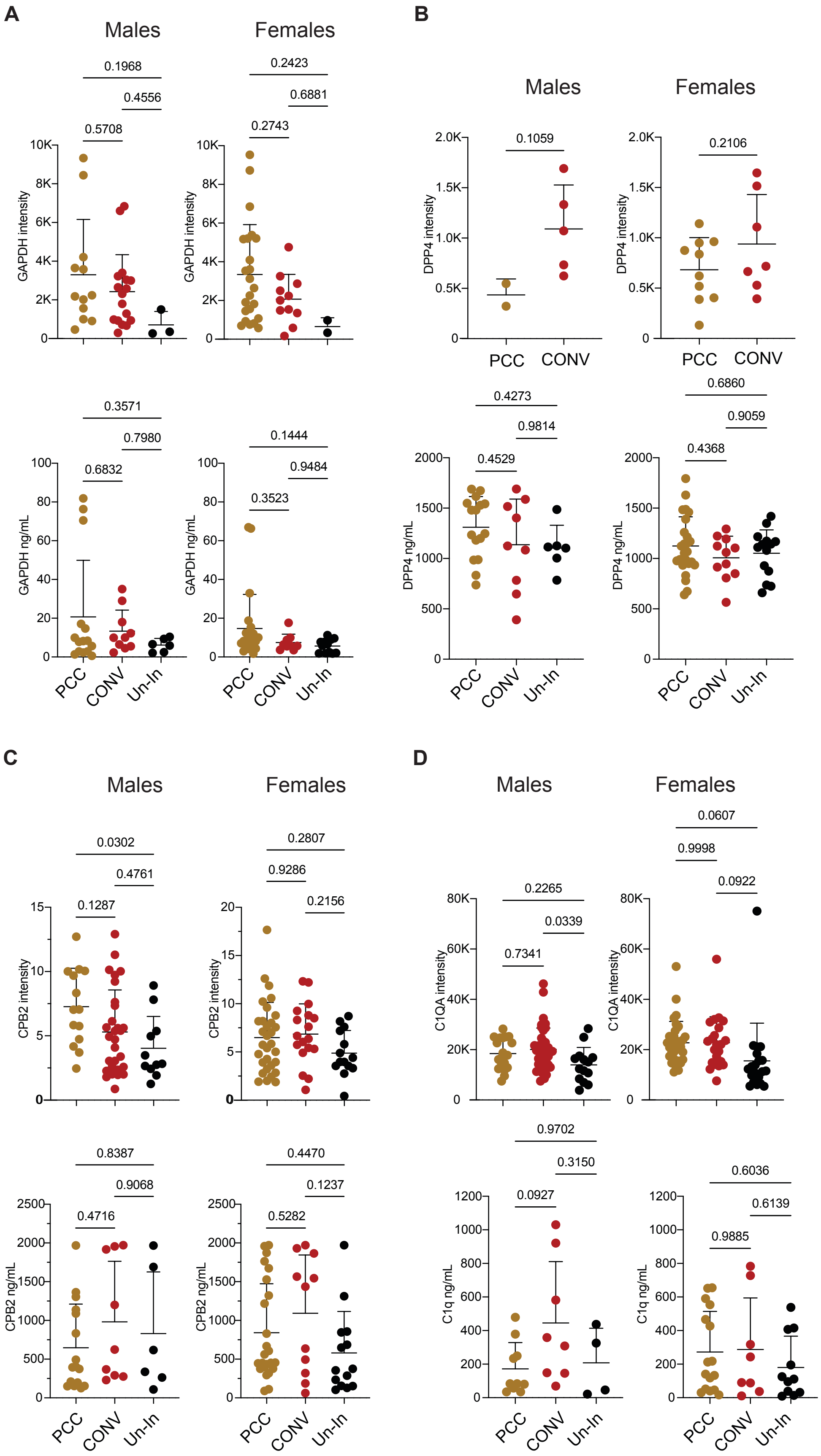

A
Males
Females
0.1968
0.4556
0.5708
GAPDH intensity
10K
8K
6K
4K
2K
0
0.2423
0.6881
0.2743
0.3571
0.7980
0.6832
GAPDH ng/mL
100
80
60
40
20
0.1444
0.9484
0.3523
PCC
CONV
Un-In
B
Males
Females
DPP4 intensity
2.0K
1.5K
1.0K
0.5K
0.1059
0.2106
PCC
CONV
0.4273
0.9814
0.4529
DPP4 ng/mL
2000
1500
1000
500
0.6860
0.9059
0.4368
C
Males
Females
0.0302
0.4761
0.1287
CPB2 intensity
15
10
5
0.2807
0.9286
0.2156
20
0.8387
0.9068
0.4716
CPB2 ng/mL
2500
2000
1500
1000
500
0.4470
0.1237
0.5282
D
Males
Females
0.2265
0.0339
0.7341
C1QA intensity
80K
60K
40K
20K
0.0607
0.9998
0.0922
0.9702
0.3150
0.0927
C1q ng/mL
1200
1000
800
600
400
200
0.6036
0.6139
0.9885

Supplementary Fig. 2: **Comparison between proteomics and ELISA measurements for a restricted set of proteins in males and females (at birth).** Peptide abundance (DIA-MS) and protein levels (ELISA) are shown for select proteins across three groups. Statistical analysis was conducted using one-way/two-way ANOVA followed by Tukey's multiple comparisons tests, with significance set at a p-value ≤ 0.05. Data are presented as mean with standard deviation.

A

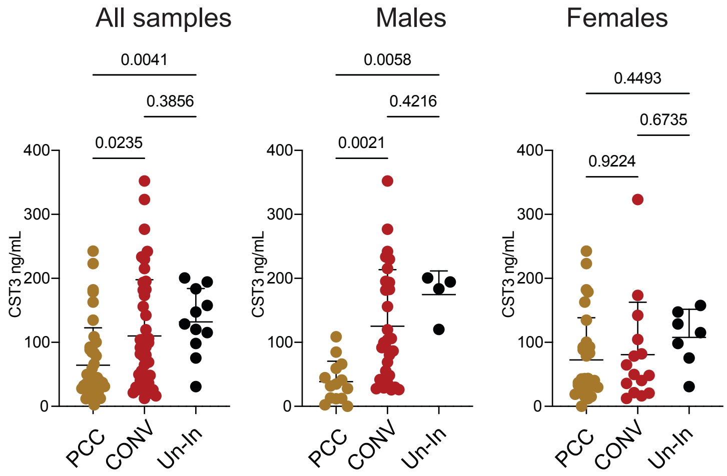

B

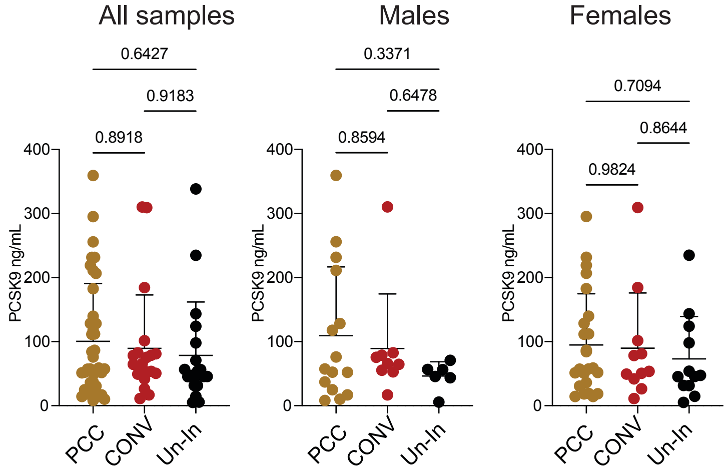

Supplementary Fig. 3: **ELISA measurements for a restricted set of proteins in all samples and following segregation into males and females (at birth).** Protein levels (ELISA) are

shown for select proteins across three groups. Statistical analysis was conducted using one-way/two-way ANOVA followed by Tukey's multiple comparisons tests, with significance set at a p-value ≤ 0.05. Data are presented as mean with standard deviation.

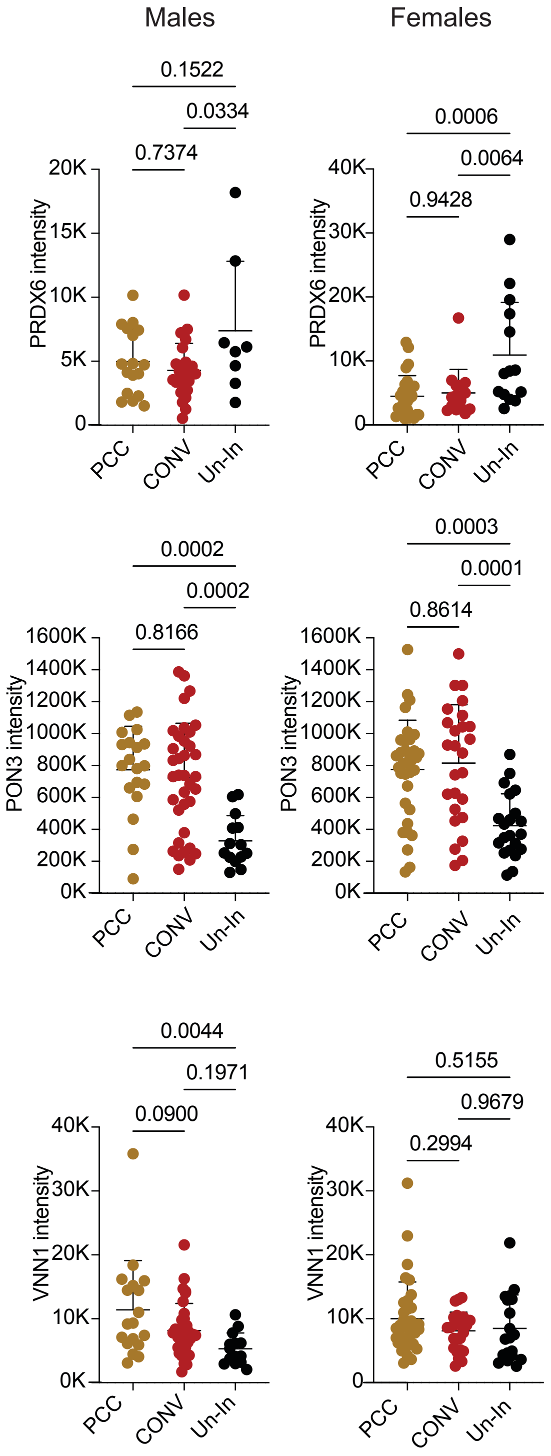

Supplementary Fig. 4: **Peptide abundance for oxidative stress related proteins in males and females (at birth).** Peptide abundance (DIA-MS) is shown for select proteins. Statistical analysis

was conducted using one-way/two-way ANOVA followed by Tukey's multiple comparisons tests, with significance set at a p-value ≤ 0.05. Data are presented as mean with standard deviation.

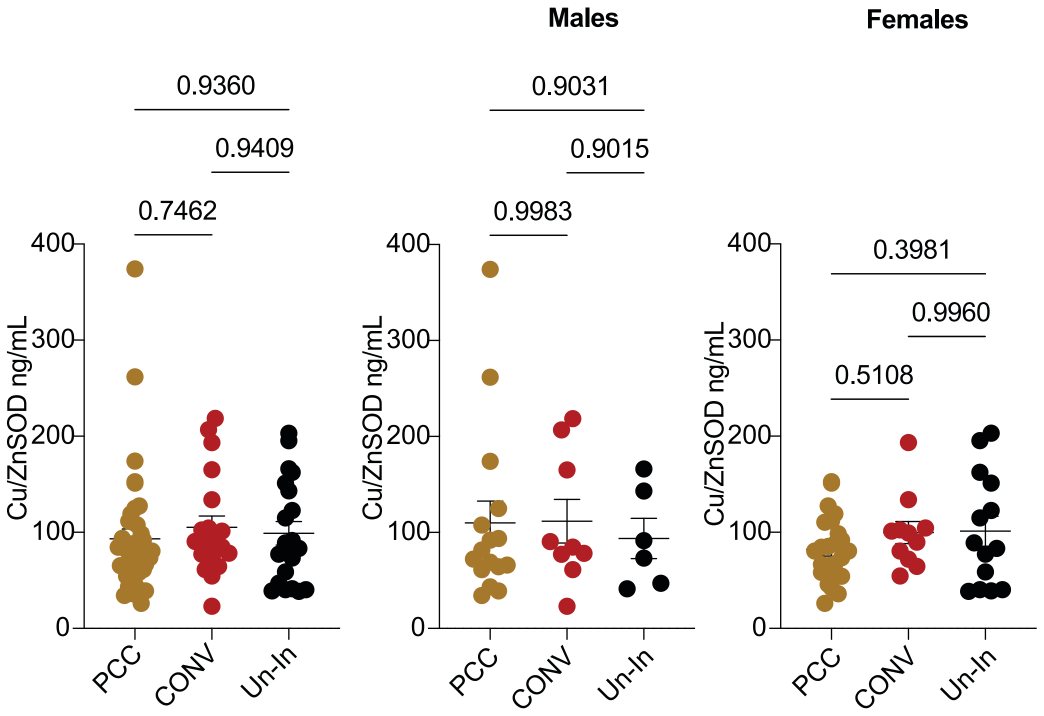

Supplementary Fig. 5: **ELISA measurements for Cu/ZnSOD.** Protein levels (ELISA) are shown in all samples and in males and females (at birth). Statistical analysis was conducted using one-way/two-way ANOVA followed by Tukey's multiple comparisons tests, with significance set at a p-value ≤ 0.05. Data are presented as mean with standard deviation.
